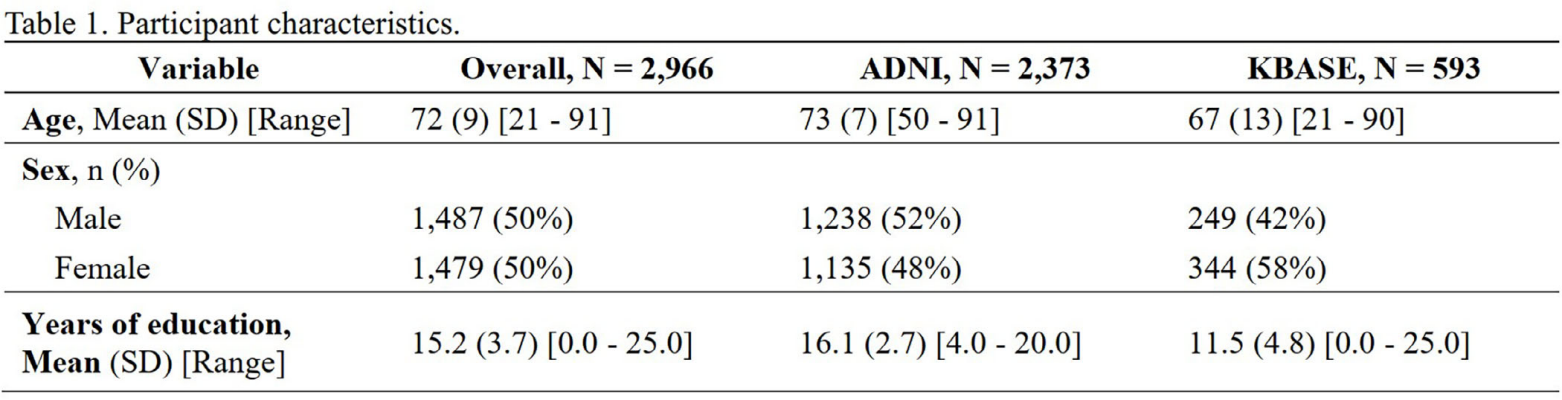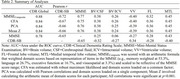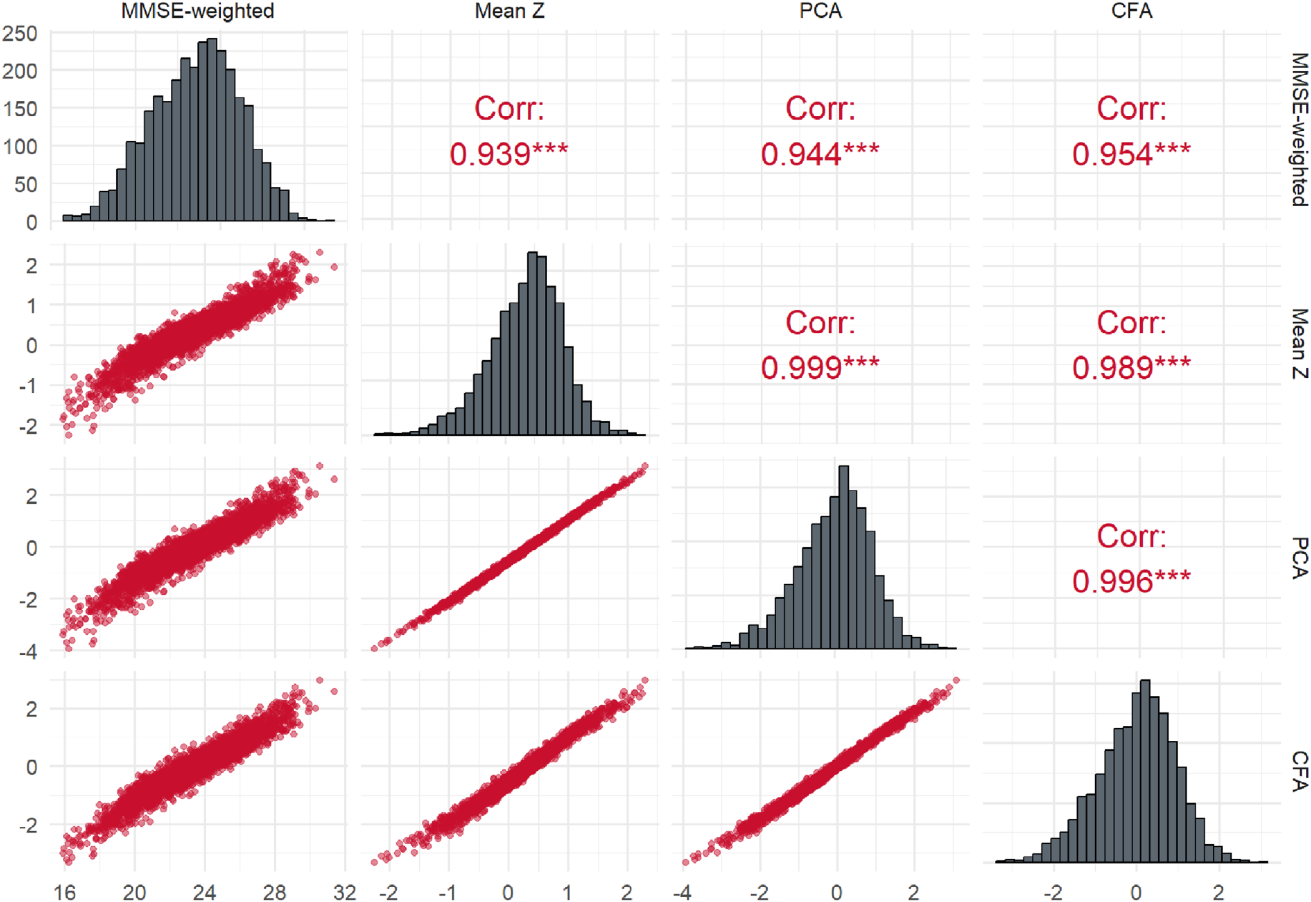# Evaluating Scoring and Weighting Methods of Global Cognition in a Harmonized United States and Korean Research Sample

**DOI:** 10.1002/alz70857_107728

**Published:** 2025-12-26

**Authors:** Joshua M. Garcia, Leslie S. Gaynor, Adam M. Staffaroni, Timothy J. Hohman, Dahyun Yi, Min Soo Byun, Dong Young Lee, Kwangsik Nho, Shannon L Risacher, Andrew J. Saykin, Meichen Yu, Evgeny J. Chumin, Luis D. Medina, Paul K Crane

**Affiliations:** ^1^ University of Houston, Houston, TX, USA; ^2^ Vanderbilt Memory & Alzheimer's Center, Vanderbilt University Medical Center, Nashville, TN, USA; ^3^ Division of Geriatric Medicine, Department of Medicine, Vanderbilt University Medical Center, Nashville, TN, USA; ^4^ Memory and Aging Center, UCSF Weill Institute for Neurosciences, University of California, San Francisco, San Francisco, CA, USA; ^5^ Vanderbilt Memory & Alzheimer's Center, Vanderbilt University Medical Center, Nashville, TN, USA; ^6^ Institute of Human Behavioral Medicine, Medical Research Center, Seoul National University, Seoul, Korea, Republic of (South); ^7^ Department of Psychiatry, Seoul National University College of Medicine, Seoul, Korea, Republic of (South); ^8^ Department of Neuropsychiatry, Seoul National University Hospital, Seoul, Korea, Republic of (South); ^9^ Indiana University School of Informatics and Computing, Indianapolis, IN, USA; ^10^ Center for Neuroimaging, Department of Radiology and Imaging Sciences, Indiana University School of Medicine, Indianapolis, IN, USA; ^11^ Indiana Alzheimer's Disease Research Center, Indiana University School of Medicine, Indianapolis, IN, USA; ^12^ Center for Neuroimaging, Department of Radiology and Imaging Sciences, Indiana University School of Medicine, Indianapolis, IN, USA; ^13^ Department of Medical and Molecular Genetics, Indiana University School of Medicine, Indianapolis, IN, USA; ^14^ Indiana Alzheimer's Disease Research Center, Indiana University School of Medicine, Indianapolis, IN, USA; ^15^ Center for Neuroimaging, Indiana University School of Medicine, Indianapolis, IN, USA; ^16^ Department of General Internal Medicine, University of Washington School of Medicine, Seattle, WA, USA

## Abstract

**Background:**

Global cognitive metrics are useful for tracking cognitive aging, yet there is no consensus on how to best define and measure global cognition. The aim of this study was to compare scoring methods for global cognition against validity indicators.

**Method:**

Using harmonized factor scores of four cognitive domains (memory, executive function, language, visuospatial) in the United States and Korea (Table 1), we compared scoring methods for global cognition with validity indicators of cognition, functioning, and structural neuroimaging at baseline. Scoring approaches included combining cognitive domains with mean z‐scoring, congeneric confirmatory factor analysis (CFA; maximum likelihood robust estimation), principal component analysis (PCA), and weighting domains based on the Mini‐Mental Status Examination (MMSE_Composite_). Correlations (*r*) were used to compare composites against continuous validity indicators. The Clinical Dementia Rating Scale Sum of Boxes (CDR‐SB) and MMSE were used to measure cognition and functioning. Neuroimaging measures included brain volume relative to cerebrospinal fluid (BV/CSF) and intracranial volume‐normalized measures of brain volume (BV/ICV), ventricular volume (VV), frontal lobe volume (FL), and medial temporal lobe volume (MTL). Receiver operating characteristic (ROC) curves assessed the ability to detect cognitive impairment (CDR‐Global≥0.5).

**Result:**

Cognitive composites were highly intercorrelated (Figure; *r* range:0.94,1.00), correlated with CDR‐SB (*r* range:‐0.72,‐0.65) and MMSE (*r* range:0.75,0.77), and detected cognitive impairment in the *good* range (AUCs=0.84‐0.88). Cognitive composites were similarly correlated with neuroimaging metrics, with differences of at most *r* = 0.02 across comparisons, except for FL, where MMSE_Composite_ had an *r* 0.04 lower than the others. Composites yielded stronger correlations with most neuroimaging metrics compared to standalone measures except for BV/ICV with CDR‐SB (*r* = ‐0.27). MTL and VV demonstrated the strongest associations with cognitive composites and other indicators of cognition/functioning.

**Conclusion:**

Regardless of scoring method, global cognitive composites were similarly related to validity indicators of cognition/functioning and structural neuroimaging in a harmonized sample. MMSE_Composite_ showed the strongest relationships with CDR but was less strongly associated with frontal lobe volume. Composites demonstrated stronger relationships with structural neuroimaging compared to standalone measures. Future work aims to evaluate similarities and differences between cohorts and evaluate relationships longitudinally.